# Effect of tetracycline on IL-1β and IL-6 levels of the peri-implant sulcular fluid

**DOI:** 10.34172/japid.2021.015

**Published:** 2021-11-17

**Authors:** Amir Mehrabi, Ramin Negahdari, Feridoun Parnia, Alireza Garjani

**Affiliations:** ^1^Department of Prosthodontics, Faculty of Dentistry, Tabriz University of Medical Sciences, Tabriz, Iran; ^2^Department of Pharmacology, Faculty of Pharmacy, Tabriz University of Medical Sciences, Tabriz, Iran

**Keywords:** Antiseptic, gingival crevicular fluid, IL-1β, IL-6, implant

## Abstract

**Background:**

Inflammation in the implant-abutment interface is one of the main factors that can reduce implant stability. Therefore, this study investigated the effect of chlorhexidine, tetracycline, saliva, and a dry environment on the interleukin IL-1β and interleukin IL-6 levels of the gingival groove fluid at the implant-abutment interface.

**Methods:**

Twenty-four (10 men and 14 women) patients referred to the Faculty of Dentistry for implant treatment, who met the inclusion criteria, were examined. Four different materials were used in each implant, including 2% chlorhexidine, 3% tetracycline, saliva, and a dry medium. Each test material was placed inside the implant screw during the anchorage session, and the healing screw was closed. Patients were then sampled in three implantation sessions and one month after prosthesis delivery. Interstitial fluid groove was used for sampling after cleaning the mouth (half an hour after three minutes of thorough brushing). The data were analyzed with SPSS 20 using ANOVA and relevant post hoc tests.

**Results:**

There was a significant difference in the mean IL-6 and IL-1β levels between the four materials (P<0.05). IL-6β levels were similar in tetracycline and chlorhexidine but significantly higher than in saliva and the dry environment (P<0.05). IL-6 and IL-1β levels in the saliva were significantly higher than in the dry environment (P<0.05).

**Conclusion:**

The use of tetracycline at the junction of implant and abutment reduces the inflammatory cytokines IL-6 and IL-1β.

## Introduction

One with a harmful effect on the implant that reduces the durability and endangers the implant’s longevity is inflammation at the implant-abutment interface.^1-3^ Microleakage at the implant-abutment interface is the most important factor in the inflammatory reaction around the implant.^4^ Microleakage causes the colonization of bacteria around the implant-abutment set, resulting in a pathophysiologic process of bone loss and subsequent loss of the implant.^5-7^

Cytokines are peptide mediators that regulate immunological responses, topical-systemic inflammatory responses, and therapeutic responses against invading factors. They bring about their effect by stimulating the proliferation and differentiation or preventing the proliferation and differentiation of cells.^8^ The most important pro-inflammatory cytokines are IL-1β and IL-6, with similar and synergic effects. IL-6 has increasingly been observed in the GCF of patients with implant treatment failure.^9,10^ IL-1β is also the main inflammatory cytokine associated with implant prostheses and is, in fact, the key molecule in the pathogenesis of implants.^11^

Dursun and Tözüm,^12^ in a meta-analysis, stated that pro-inflammatory cytokines in the crevicular fluid surrounding the implant indicate the condition of the disease or health around the implant. Maheshwari et al^13^ showed differences in IL-1β levels in different stages of implant delivery. However, Nogueira-Filho et al^14^ concluded that interleukin levels of 4.6 and 10 in the fluid around the implant and gingival crevicular fluid of healthy teeth were not significantly different from each other and did not change over time.

Controlling diseases around the implant is an unpredictable and challenging task. One of the most important factors in treating similar conditions in natural teeth, preventing biofilm development and removing it from the implant surface, should be the first step in maintaining the health of soft tissues around the implant. Combination therapies, including mechanical debridement, have been studied with due observance not to harm the hemidesmosome junctions in the sulcus base, along with antimicrobial factors such as chlorhexidine and essential oils, have shown effective and satisfactory results.^15-17^

Tetracycline is a bacteriostatic antibiotic. Bacteriostatic antibiotics affect the proliferation and growth of bacteria and, by reducing bacterial reproduction, provide the host’s defense with the opportunity to kill bacteria. This antibiotic is used more than any other antibiotics in severe destructive and progressive periodontitis and in cases not responding to common treatments.^18^

Considering the paucity of studies on the effect of antiseptics on inflammatory factors at the implant-abutment interface, this study was conducted to investigate the effect of four substances, chlorhexidine, tetracycline, saliva, and a dry environment, on the levels of pro-inflammatory cytokines IL-1β and interleukin IL-6 in the gingival crevicular fluid at the implant-abutment interface.

## Methods

In this split-mouth study, 24 patients referring to the Faculty of Dentistry, Tabriz University of Medical Sciences, for an implant treatment were examined. The inclusion criteria for the study were ≥18 years of age, having at least four implants in one jaw, and a probing depth of about 1-3 mm around the implant. Patients undergoing scaling in the last three months, pregnant and breastfeeding women, patients receiving antibiotics in the last six months, those with systemic diseases such as diabetes that affected oral health, drug addicts, smokers, alcohol users, those with a history of bisphosphonate use, and those taking systemic anti-inflammatory drugs were excluded.

Osstem implant system (TS, OSSTEM, Huntingdon, Cambridgeshire) was used for each patient. Each implant in each patient comprised four different substances (2% chlorhexidine, 3% tetracycline, saliva, and a dry environment).

Each material examined in the uncovering session was placed inside the implant screw, and the healing screw was closed. Then, in three sessions of prosthesis delivery molding and one month after the prosthesis delivery, the patients underwent a sampling procedure.

The gingival crevicular fluid was used for sampling after oral cleansing (half an hour after three minutes of thorough brushing). Each area was isolated by cotton rolls and air-dried for 5 seconds to remove any salivary contamination from the mentioned area. The samples were obtained by paper points (Meta Biomed Co., Ltd, Chungbuk, Korea). These paper points were placed in four distinct regions (mesiobuccal, distobuccal, mesiolingual, and distolingual) in the gingival crevice to the extent that medium resistance was felt and kept there for 30 seconds. Paper points contaminated with blood were excluded. Then each paper point was placed in a small sterile tube and frozen at -80°C until all the samples were prepared. The samples were sent to the immunology laboratory to determine the pro-inflammatory cytokines IL6 and IL1β by an ELISA kit.19 The data were analyzed with SPSS 20 using ANOVA.

## Results

In this study, 24 patients with at least four implants in one jaw were examined; 58.3% (14 patients) were female, and 41.7% (10 patients) were male. According to ANOVA, there were significant differences in the mean IL-6 and IL-1β levels between the four materials substances (P<0.05). Table 1 presents the comparison of IL-6 and IL-1β cytokine levels between the four materials.

**Table 1:** Comparison of IL-6 and IL-1β cytokine levels between the four materials

**Sample**	**Number**	**IL-6**		**IL-1β**	
		**Mean**	**SD**	**Mean**	**SD**
**Tetracycline**	24	14.93	1.845	12.20	1.565
**Chlorhexidine**	24	14.36	1.572	11.33	2.417
**Saliva**	24	18.08	2.805	14.83	2.140
**Dry environment**	24	16.04	2.694	13.83	2.099
**P-value** ^ 2 ^		P≤0.001		P≤0.001	

^1 ^P-value: One-way ANOVA

According to post hoc Tukey tests presented in Table 2, pair-wise comparisons of the materials showed that IL-6 and IL-1β levels in the tetracycline and chlorhexidine groups were similar. IL-6 and IL-1β levels in the tetracycline and chlorhexidine groups were significantly higher than in saliva and dry environment. IL-6 and IL-1β levels in the saliva were significantly higher than in the dry environment.

**Table 2:** Comparison of chlorhexidine and tetracycline in terms of IL6 and IL1β

**Sample 1**	**Sample 2**	**IL-6**		**IL-1β**	
		**Average difference (I-J)**	**P-value**	**Average difference (I-J)**	**P-value** ^ 1 ^
**Tetracycline**	**Chlorhexidine**	0.56667	0.827	0.870	0.471
**Tetracycline**	**Saliva**	-3.15417^*^	≤0.001	-2.625^*^	≤0.001
**Tetracycline**	**Dry environment**	-1.10833	0.343	-1.625^*^	0.040
**Chlorhexidine**	**Saliva**	-3.72083^*^	≤0.001	-3.495^*^	≤0.001
**Chlorhexidine**	**Dry environment**	-1.67500	0.042	-2.495^*^	≤0.001
**Saliva**	**Dry environment**	2.04583^*^	0.014	1.00000	0.347

^1^P-value: Tukey HSD

## Discussion

The IL-1β level was significantly different between the four study groups so that in the chlorhexidine (11.33±2.41) and tetracycline (12.20±1.5) groups, it was significantly lower than the saliva (14.83±2.14) and the dry environment (13.83±2.9) groups. IL-1β levels were similar in chlorhexidine and tetracycline groups. Also, the IL-1β level was higher in the saliva group than in the dry environment group, but the difference was not significant. Studies on implant-induced gingival inflammation have shown that inflammation activates intrinsic immune receptors, affecting the expression of the pro-inflammatory cytokines.^20^ Yaghobee et al^21^ showed that the IL-1β level in the peri-implant gingival crevicular fluid was significantly higher than healthy implants and healthy teeth. Wang et al^22^ reported that the IL-1β levels in a healthy implant were significantly lower than in peri-implantitis. In addition, the IL-1β with an odds ratio of OR=7.71 exhibited the highest detection capability to predict the disease condition.

In the oral cavity, local cells of the connective tissue (fibroblasts and endothelial cells) form IL-1β, or they are released from leukocytes such as mononuclear cells, macrophages, and polymorphonuclear cells.^23^ IL-1β results in the widespread expression of the cyclooxygenase 2 gene to produce nitric oxide synthase and metalloproteinase matrix.^24^ These enzymes themselves activate osteoclasts and bone erosion and collapse type 1 collagen in the bone.^25^ IL-1β is more important in the stimulation of bone absorption and is an isoform of IL-1 which is more common in periodontitis.^26^

To reduce the patient’s inflammatory response and reduce the deterioration of the peri-implant bone during the insertion of the healing abutment, Sinjari et al^27^ recommended an anesthetic protocol with 0.2% chlorhexidine gel from the time of implant insertion to crown delivery. The researchers showed that the use of CHX gel in the interface caused significant bone loss during the first year. de Waal et al^28^ showed that the disinfection of the implant surface with 0.12% chlorhexidine in the surgical treatment of peri-implantitis led to faster anaerobic bacteria removal from the implant surface than the placebo solution.

Chlorhexidine is the most effective ingredient in removing microbial flora, reducing plaque and gingival inflammation.^29^ The mechanism of this effect involves reducing the formation of pellicles, change in bacterial adhesion to dental surfaces, and change in the bacterial cell wall, and the subsequent bacterial death.^29^

Xu et al^30^ showed that topical use of antibiotics and systemic antibiotics could reduce inflammatory responses in wound healing after implant surgery.

Tetracycline reduces infections and inflammation and ultimately reduces the number of inflammatory cytokines. In the present study, IL-1β levels were similar in both chlorhexidine and tetracycline groups, indicating the same function of these two cytokines. Tetracyclines mainly perform their antibacterial activity by inhibiting the synthesis of microbial proteins. In a study, researchers investigated the potential of tetracycline for disinfection. Samples were washed with 50 mL/mL tetracycline under osteotomy implant surgery and reported that tetracycline reduced Streptococcus counts seven-folds.^26^ In a study on the effect of topical tetracycline delivery on the treatment of peri-implantitis, researchers treated 30 lesions with mechanical lesions and insertion of fiber tetracycline and reported significant improvements in clinical parameters.^31^

In the present study, IL-6 levels were significantly different between the four study groups. In the chlorhexidine (14.36±1.57) and tetracycline (14.93±1.84) groups, it was significantly lower than the saliva (18.08±2.80) and the dry environment (16.04±2.69) groups. Also, IL-6 levels were significantly higher in the saliva group than in the dry environment group.

Yaghobee et al^21^ reported a significant difference in the IL-6 level in the gingival crevicular fluid in peri-implantitis and healthy implants and between peri-implantitis and healthy teeth. Liskmann et al^32^ introduced IL-6 as an effective representative for the diagnosis of diseases of implant prosthesis, confirming its impact on assessing the ability of the immune system to achieve an inflammatory balance. In these researchers’ studies, the IL-6 level was significantly higher in healthy implants compared to peri-implants.

IL-6 is a multi-functional cytokine that regulates immune responses and acute phase reactions, with a crucial role in the host defense mechanism and inflammatory and immune responses. IL-6 is not permanently produced by normal cells, but its expression is increased by a variety of cytokines, lipopolysaccharides, or viral infections.^33^

Abraham et al^29^ showed that chlorhexidine is an effective antiseptic in the prevention and management of peri-implantitis. Park^34^ evaluated the use of tetracycline in a 48-year-old male with peri-implantitis. After disinfecting the defect area with the mixture at a 4:1 ratio, the bone was proteinized, and tetracycline was transplanted. The results showed the recovery of soft tissue without interruption.

Tetracycline is a bacteriostatic antibiotic. In a study on dogs, the use of tetracycline solution resulted in re-osseointegration. In this study, it was shown that when bone defects around implants are cleaned with tetracycline, re-osseointegration occurs at a rate of 1.77 mm in four months.^35^

One of the reasons for the use of antibiotics is in various treatments is chlorhexidine toxicity. Chlorhexidine cytotoxicity in human bone cells has been studied. Cytotoxicity appears to be affected by concentration and time. SEM analysis confirmed the absence of osteoblast phenotype changes after exposure to 0.2% CHX for 1 minute and 1% CHX for 30 seconds.35s.

## Conclusions

Based on the results, it can be concluded that tetracycline at the junction of implant-abutment reduces the inflammatory cytokines IL-6 and IL-1β. The results of the present study revealed that inflammation around the implant could be managed with antiseptic agents. This can enhance the stability of the implants. Besides, it can prevent the development of biofilm and save the health of soft tissues around the implant.

## Authors’ contributions

RN, FP, and AG designed the study. AM carried out the study procedures. All authors contributed to the manuscript writing and its critical revision and read and approved the final manuscript.

## Availability of data

The raw data from the reported study are available upon request from the corresponding author.

## Ethics approval

The protocol of the present study was approved by the Ethics Committee of Tabriz University of Medical Sciences under the code IR.TBZMED.REC.1398.166. Written informed consent was obtained from all the patients.

## Competing interests

The authors declare that they have no competing interests with regard to the authorship and/or publication of this paper.
